# The use of the ratio of C-reactive protein to albumin for the diagnosis of pediatric septic arthritis

**DOI:** 10.3389/fped.2023.1308513

**Published:** 2024-01-16

**Authors:** Chong Ren, Quanwen Yuan, Chunhua Yin, Feng Yao, Wentao Yu, Fuyong Zhang, Xiaodong Wang

**Affiliations:** ^1^ Department of Orthopedics, Children’s Hospital of Soochow University, Suzhou, Jiangsu, China; ^2^ Department of Orthopedics, Guiyang Maternal and Child Health-Care Hospital, Guiyang, Guizhou, China

**Keywords:** c-reactive protein, albumin, diagnosis, pediatric, septic arthritis

## Abstract

**Purpose:**

This study aimed to investigate the relationship between the ratio of c-reactive protein to albumin (CAR) and pediatric septic arthritis (PSA).

**Methods:**

Clinical and laboratory data were collected. Receiver operating characteristic (ROC) curve analysis was used to evaluate the predictive ability of CAR in identifying PSA. Multivariable logistic regression analyses was performed to calculate adjusted odds ratio (OR) with 95% confidence interval (CI).

**Results:**

We included 305 patients with PSA (CAR ≤ 0.447, 182 patients; CAR > 0.447, 123 patients) between September 2013 and November 2022. ROC analysis showed that CAR performed best in diagnosing PSA, with an area under curve (AUC) value of 0.828. After adjusted for potential confounders, we found that high CAR was associated with PSA (OR = 6.85, 95% CI: 2.30–20.40, *p* = 0.001). In sensitivity analyses, subgroups analyses, and propensity score matching, the results remain stable.

**Conclusions:**

The CAR (>0.447) at admission was an independent risk factor for PSA. It is worthy to further investigate this association.

## 1 Introduction

Pediatric septic arthritis (PSA) is a severe infection with high incidence of local and systemic complications, particularly in growing children (1–3). These may be initially absent at PSA onset time causing a delay in diagnosis and treatment which may lead to permanent sequelae (4–7). The optimum diagnostic strategy for the early detection of PSA may reduce not only the number of unnecessary surgical operations but also the risk of complications and may contribute significantly to reducing hospital stays and costs to the family. Presenting clinical features, imaging studies and laboratory tests may be helpful in the diagnosis of PSA, but early diagnosis remains a challenge (8). There are several techniques to diagnose PSA at early stages, such as magnetic resonance imaging (MRI), ultrasound and joint puncture; nevertheless, these techniques may be limited in rural and remote areas owing to a lack of medical technicians to carry them out. Furthermore, MRI usually requires general anesthesia in very young patients. Consequently, simple, less-invasive, and low-cost routine detection methods to estimate PSA are currently of interest.

C-reactive protein (CRP) is a positive acute phase protein produced in the liver following a cytokine-induced stimulation as a result of ischemia, trauma, or inflammation (9). CRP plays a regulatory role in the inflammatory process by activating complement and enhancing the function of phagocytes (10). Albumin (ALB), as a negative acute phase reactant synthesized by the liver, decreases during inflammation, and is negatively interrelated with inflammation severity, disease prognosis and mortality (11). Systemic inflammation can reduce serum albumin concentrations by increasing capillary permeability (12). The ratio of CRP to albumin (CAR) is a new score based on nutrition and inflammation, reflecting not only nutritional status but also systemic inflammation status. Previous studies have shown that CAR have certain values for the early diagnosis of nosocomial infection (10, 13, 14). In recent years, many studies have shown that CAR can indicate the degree of inflammation and prognosis in pancreatitis and neonatal septicaemia (15, 16). Moreover, CAR is used as an important prognostic factor for many malignancies, such as pancreatic cancer and esophageal cancer (17, 18).

To our best knowledge, there is currently no study investigating the role of CAR in predicting PSA. In the present study, we aim to investigate the predictive significance of CAR for the diagnosis of PSA, using the multivariate logistic regression analysis adjusting for a range of confounders.

## 2 Participants and methods

### 2.1 Study population

The study was conducted according to the Declaration of Helsinki guidelines and approved by the Medical Ethics Committee of Children's Hospital of Soochow University. The records of pediatric patients followed with suspected septic arthritis at our hospital from September 2013 to November 2022 were obtained from the electronic medical record system and reviewed. A total of 305 pediatric patients were enrolled in this study. The information of these patients was anonymous with no identifiable information recorded and maintained with confidentiality. Patients with the following conditions were included: (1) aged <16 years; (2) pediatric patients with suspected septic arthritis. Patients who met the following criteria were excluded: (1) missing critical data (including CRP and albumin); (2) undiagnosed case at discharge; (3) infections of the craniofacial area, ribs and spine.

### 2.2 Definition

Septic arthritis was defined on the basis of synovial white cell count and culture (8). Septic arthritis was defined as either (1) a positive culture from a joint or (2) positive blood cultures with a synovial fluid aspirate that was grossly purulent or with a white blood cell count of greater than 50,000/µl (19).

### 2.3 Data collection

The demographic data and laboratory indicators of patients were collected from the electronic medical system. The baseline characteristics were collected from the patients' medical records, including age, gender, onset time, onset site, and diagnosis. The onset time was days from the first symptom onset date until the date of admission. For subjects with multiple onset sites, only the first site of onset was used for analysis. The onset site was classified as hip, knee, and any other site (denoted as “other”). The laboratory tests at admission, such as white blood cell count (WBC), CRP, haemoglobin, erythrocyte sedimentation rate (ESR), mean platelet volume (MPV), platelet count (PLT), neutrophil count (NEUT), lymphocyte count (LYMPH), monocytes count (MONO), albumin, and globulin were collected and evaluated from the electronic medical records. Of all the variables, only 29 (9.5%) were missing ESR data. It is worth noting that all venous blood samples were collected routinely by nurses within 24 h after the first admission and sent to the clinical laboratory of our institution for testing.

The indexes were calculated according to the following equations: (1) neutrophil to lymphocyte ratio (NLR) = neutrophil counts (×10^9^/L)/lymphocyte counts (×10^9^/L); (2) platelet to lymphocyte ratio (PLR) = platelet counts (×10^9^/L)/lymphocyte counts (×10^9^/L); (3) platelet to MPV ratio (PVR) = platelet counts (×10^9^/L)/MPV (fL); (4) albumin to globulin ratio (AGR) = albumin (g/L)/globulin (g/L); (5) CRP to albumin ratio (CAR) = CRP (mg/L)/albumin (g/L).

### 2.4 Statistical analysis

Descriptive statistical analysis was applied to all participants' data. Continuous variables were expressed as mean ± standard deviation (SD), and categorical variables were expressed as frequency (%). Missing data were interpolated using the average value. The independent sample *t*-test was used to compare normally distributed quantitative data and the Mann-Whitney *U* test was used for non-normally distributed variables. Categorical data were compared using the Chi-square test. Spearman's correlation test was used to assess correlation. The diagnostic performance levels of the different biomarkers were compared based on the results of receiver operating characteristic (ROC) analysis, calculating area under curve (AUC), sensitivity, specificity, the positive predictive value (PPV), and the negative predictive value (NPV). The optimal cutoff was determined by the Youden index. Significant factors were included in the stepwise multivariate logistic regression model, and independent predictor was identified. All the analyses were performed with the statistical software packages R version 4.1.1 and Free Statistics software versions 1.8. A *p* value of <0.05 was considered statistically significant.

### 2.5 Sensitivity analyses

To ensure that our findings were robust, we performed a propensity score matching (PSM). Patients with missing data were excluded and a 1: 1 nearest-neighbor matching algorithm was applied. The variables selected for the propensity score model were as follows: age, gender, onset time, WBC, ESR, globulin, NEUT, LYMPH, MONO, haemoglobin, PLT, MPV, AGR, NLR, PLR, and PVR. The PSM degree was estimated by a standardized mean difference (SMD). A threshold <0.1 was considered acceptable.

## 3 Results

### 3.1 Baseline characteristics

**Figure 1:**
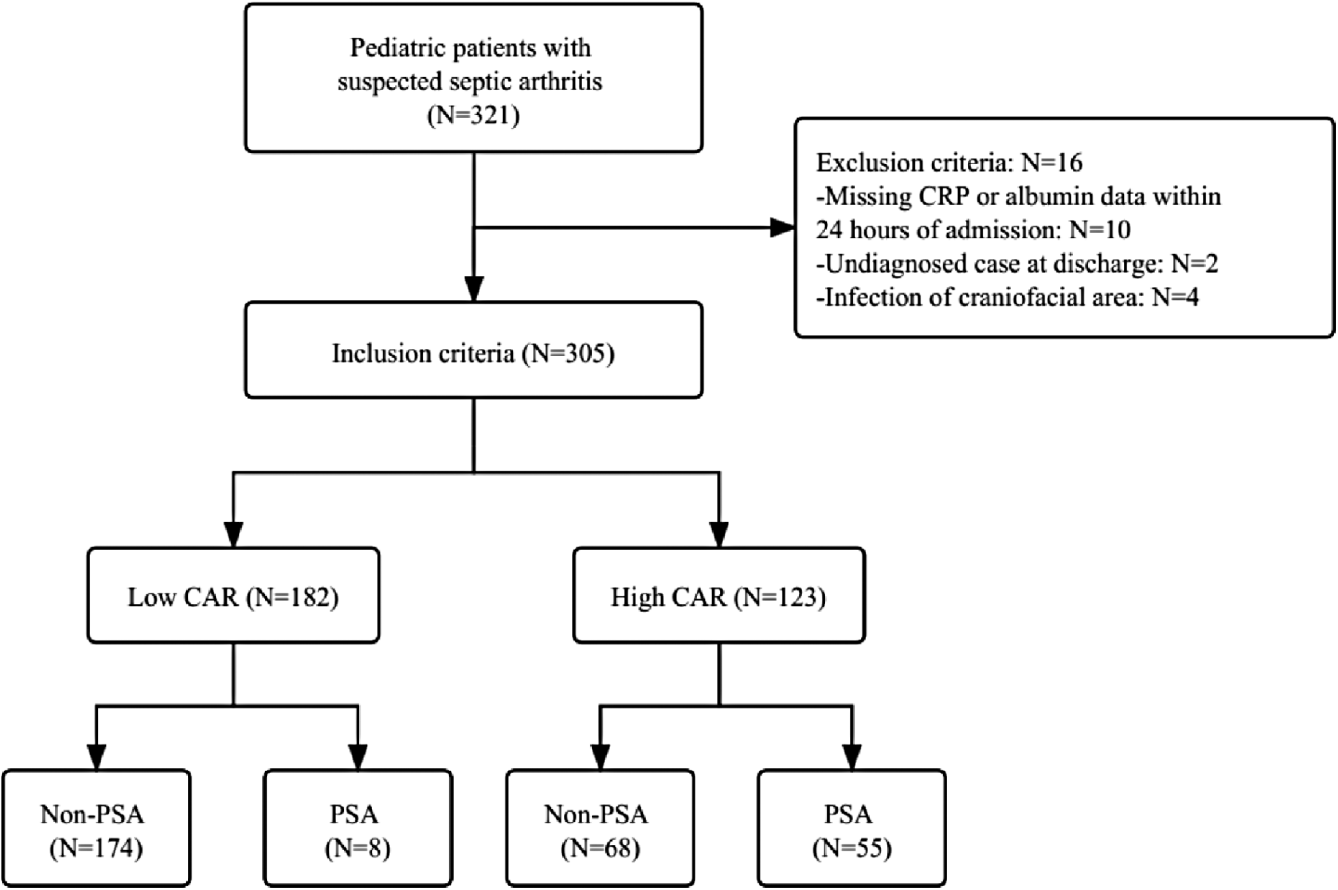
The flow chart of the study. CRP, c-reactive protein; CAR, c-reactive protein to albumin ratio; PSA, pediatric septic arthritis.

We included 305 patients in our study: 182 low CAR (59.7%) and 123 high CAR (40.3%). The flow chart of the study participants is presented in Figure 1.

**Figure 2:**
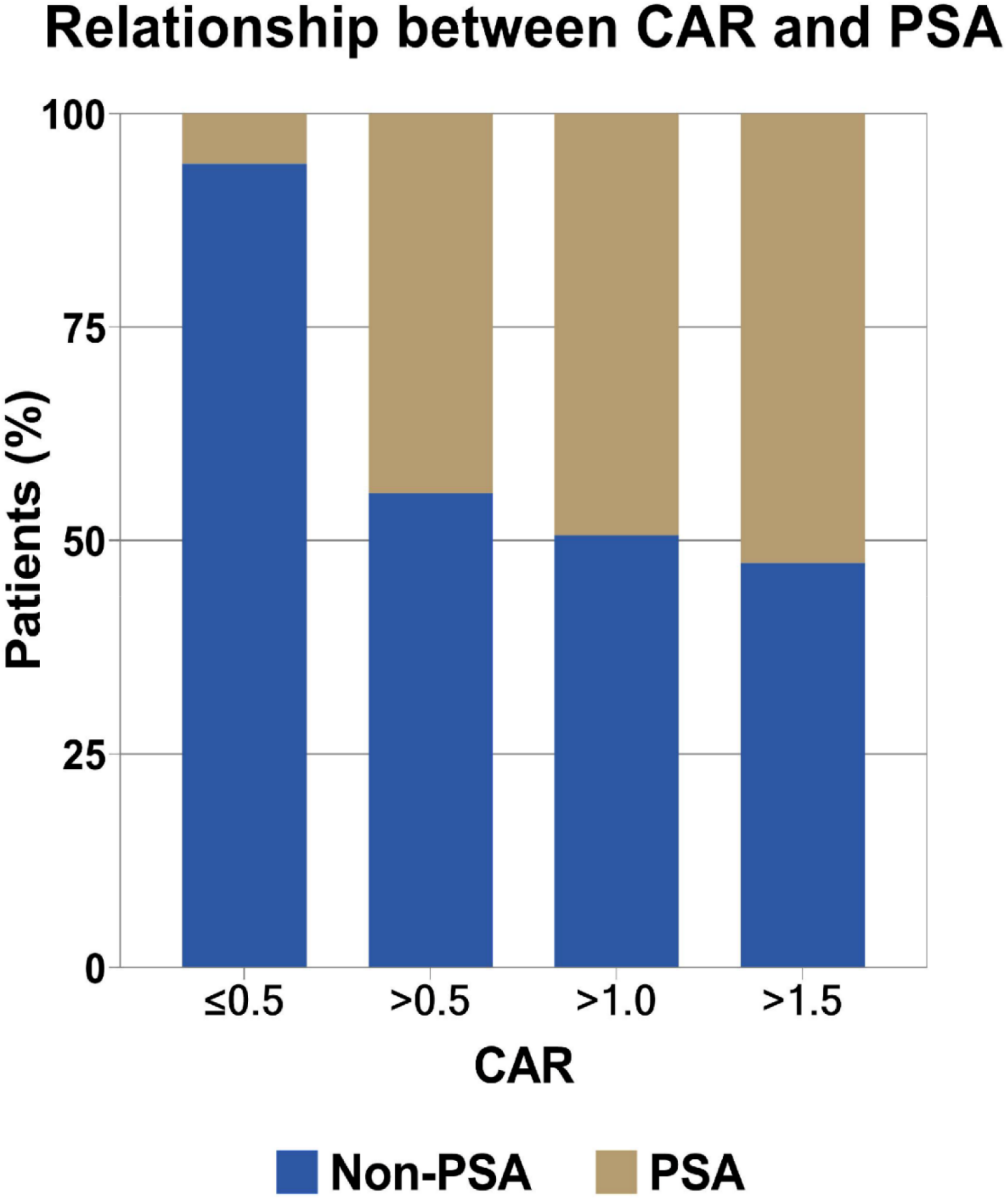
Relationship between CAR and PSA.

ROC analyses (Table 1) indicated that CAR performed best in diagnosing PSA (AUC: 0.828, 95% CI: 0.777–0.879; optimal cutoff: 0.447), followed by CRP (AUC: 0.824, 95% CI: 0.772–0.875; optimal cutoff: 20.835 mg/L) and ESR (AUC: 0.813, 95% CI: 0.756–0.871; optimal cutoff: 26.353 mm/h). Our further analysis indicated that the optimal cutoff value was 0.447 for CAR, and this led to a sensitivity of 87.30%, specificity of 71.90%, positive predictive value (PPV) of 44.72%, and negative predictive value (NPV) of 95.60%. Furthermore, the risk of PSA incidence increased with higher CAR, as shown in Figure 2.

**Table 1:** Performance of different individual biomarkers for the diagnosis of PSA.

Variables	AUC (95% CI)	Optimal cutoff	Sensitivity (%)	Specificity (%)	PPV (%)	NPV (%)
CAR	0.828 (0.777–0.879)	0.447	87.30	71.90	44.72	95.60
CRP (mg/L)	0.824 (0.772–0.875)	20.835	87.30	71.90	44.72	95.60
ESR (mm/h)	0.813 (0.756–0.871)	26.353	85.71	68.60	41.54	94.86
MONO (×10^9^/L)	0.809 (0.752–0.867)	0.765	80.95	71.90	42.86	93.55
Hemoglobin (g/L)	0.786 (0.726–0.847)	119.5	80.95	69.42	40.80	93.33
Albumin (g/L)	0.740 (0.671–0.810)	43.45	77.78	60.74	34.03	91.30
WBC (×10^9^/L)	0.718 (0.647–0.790)	11.885	65.08	71.49	37.27	88.72
Age (years)	0.708 (0.622–0.794)	2.75	57.14	84.71	49.32	88.36
AGR	0.706 (0.636–0.775)	1.714	77.78	61.16	34.27	91.36
NEUT (×10^9^/L)	0.662 (0.584–0.741)	7.105	60.32	69.01	33.63	86.98
PLT (×10^9^/L)	0.651 (0.561–0.742)	477	44.44	92.56	60.87	86.49
PVR	0.648 (0.559–0.737)	49.08	44.44	89.26	51.85	86.06
Globulin (g/L)	0.594 (0.512–0.675)	25.75	60.32	62.81	29.69	85.88
LYMPH (×10^9^/L)	0.594 (0.503–0.686)	3.78	52.38	72.73	33.33	85.44
NLR	0.565 (0.479–0.652)	3.131	41.27	76.03	30.95	83.26
Onset time (days)	0.513 (0.440–0.586)	2.5	80.95	33.88	24.17	87.23
PLR	0.499 (0.416–0.582)	103.158	52.38	55.37	23.40	81.71
MPV (fl)	0.482 (0.404–0.560)	8.85	87.30	16.12	21.32	82.98

PSA, pediatric septic arthritis; AUC, area under the curve; PPV, positive predictive value; NPV, negative predictive value; CAR, c-reactive protein to albumin ratio; CRP, c-reactive protein; ESR, erythrocyte sedimentation rate; MONO, monocytes count; WBC, white blood cell count; AGR, albumin to globulin ratio; NEUT, neutrophil count; PLT, platelet count; MPV, mean platelet volume; PVR, platelet to MPV ratio; LYMPH, lymphocyte count; NLR, neutrophil to lymphocyte ratio; PLR, platelet to lymphocyte ratio.

### 3.2 The optimal cut–off values of CAR and associations with clinical variables

The 305 eligible patients were divided into two groups based on the optimal cut-off value of CAR (Supplementary Figure S1). A total of 182 patients (59.7%) with CAR ≤ 0.447 on admission were defined as the low CAR group, and the rest of the patients (*n* = 123, 40.3%) with CAR > 0.447 on admission were defined as the high CAR group. The baseline characteristics of all participants were listed in Table 2. The study participants consisted of 199 (65.2%) males and 106 (34.8%) females. Overall occurrence of PSA was 20.7% (63 out of them 8 were low CAR and 55 were high CAR) and 79.3% (242 out of them 174 were low CAR and 68 were high CAR) subjects was non-PSA. The mean age of all 305 subjects was 5.7 ± 3.6 years. The mean age for low CAR was 6.1 ± 3.3 years and the mean age for high CAR was 5.0 ± 4.0 years. The onset sites included 192 (63.0%) hips, 71 (23.3%) knees and 42 (13.8%) other sites and three patients had multiple sites involved. The incidence of PSA significantly increased (*p* < 0.001) in patients with high CAR (*n* = 55, 44.7%) compared to those with low CAR (*n* = 8, 4.4%).

**Table 2:** Baseline characteristics of patients.

Variables	All patients	Low CAR	High CAR	*p* value
Patients, *n*	305	182	123	
Age (years)	5.7 ± 3.6	6.1 ± 3.3	5.0 ± 4.0	0.011
≤3	81 (26.6)	31 (17)	50 (40.7)	<0.001
>3–6	106 (34.8)	75 (41.2)	31 (25.2)	
>6	118 (38.7)	76 (41.8)	42 (34.1)	
Gender, *n* (%)				
Male	199 (65.2)	124 (68.1)	75 (61)	0.198
Female	106 (34.8)	58 (31.9)	48 (39)	
Onset time (days)	6.2 ± 5.6	6.8 ± 6.2	5.4 ± 4.2	0.035
≤3	133 (43.6)	79 (43.4)	54 (43.9)	0.026
>3–7	88 (28.9)	44 (24.2)	44 (35.8)	
>7	84 (27.5)	59 (32.4)	25 (20.3)	
Onset site, *n* (%)				
Hip	192 (63.0)	127 (69.8)	65 (52.8)	0.002
Knee	71 (23.3)	39 (21.4)	32 (26.0)	
Other	42 (13.8)	16 (8.8)	26 (21.1)	
WBC (×10^9^/L)	11.7 ± 5.5	10.2 ± 3.9	14.1 ± 6.6	<0.001
CRP	33.6 ± 49.5	4.8 ± 5.6	76.0 ± 54.9	<0.001
ESR (mm/h)	26.7 ± 27.7	13.8 ± 17.8	45.8 ± 28.6	<0.001
Albumin (g/L)	43.3 ± 3.7	44.5 ± 2.7	41.4 ± 4.2	<0.001
Globulin (g/L)	25.7 ± 4.3	25.1 ± 4.0	26.5 ± 4.5	0.004
NEUT (×10^9^/L)	7.2 ± 4.7	5.8 ± 3.4	9.3 ± 5.6	<0.001
LYMPH (×10^9^/L)	3.5 ± 1.9	3.4 ± 1.6	3.5 ± 2.3	0.650
MONO (×10^9^/L)	0.8 ± 0.5	0.6 ± 0.3	1.1 ± 0.7	<0.001
Hemoglobin (g/L)	121.7 ± 12.3	126.2 ± 9.7	115.0 ± 12.6	<0.001
PLT (×10^9^/L)	353.5 ± 123.3	334.1 ± 98.9	382.3 ± 148.3	<0.001
MPV (fl)	9.8 ± 1.0	9.8 ± 1.0	9.8 ± 0.9	0.602
AGR	1.7 ± 0.3	1.8 ± 0.3	1.6 ± 0.3	<0.001
NLR	3.0 ± 3.7	2.3 ± 3.2	4.0 ± 4.2	<0.001
PLR	124.6 ± 73.9	113.4 ± 58.6	141.3 ± 89.7	0.001
PVR	37.0 ± 14.8	34.9 ± 12.6	40.1 ± 17.1	0.002
Discharge diagnosis				
Non-PSA	242 (79.3)	174 (95.6)	68 (55.3)	<0.001
PSA	63 (20.7)	8 (4.4)	55 (44.7)	

The results of univariate logistic analysis revealed that high CAR [odds ratio (OR) = 17.59, 95% confidence interval (CI): 7.96–38.87, *p* < 0.001] was significantly associated with PSA. The multivariate logistic analyses showed that high CAR (OR = 6.85, 95% CI: 2.30–20.40, *p* = 0.001) was independent predictor of PSA. In the extended multivariable logistic regression models (Table 3), we observed that the odds ratios (ORs) of high CAR were consistently significant in all five models (ORs range: 5.98–17.59, *p* < 0.05 for all).

**Table 3:** Association between high CAR and PSA using an extended model approach.

Model	Odds ratio of high CAR	95% confidence interval	*p* value
Model 1	17.59	(7.96, 38.87)	<0.001
Model 2	14.42	(6.40, 32.47)	<0.001
Model 3	13.54	(5.94, 30.88)	<0.001
Model 4	5.98	(2.13, 16.78)	0.001
Model 5	6.85	(2.30, 20.40)	0.001

Adjusted covariates: Model 1 = CAR. Model 2 = Model 1+ (age, gender). Model 3 = Model 2+ (onset time, onset site). Model 4 = Model 3+ (WBC, CRP, ESR, albumin, globulin, NEUT, LYMPH, MONO, hemoglobin, PLT, MPV). Model 5 = Model 4+ (AGR, NLR, PLR, PVR).

**Figure 3:**
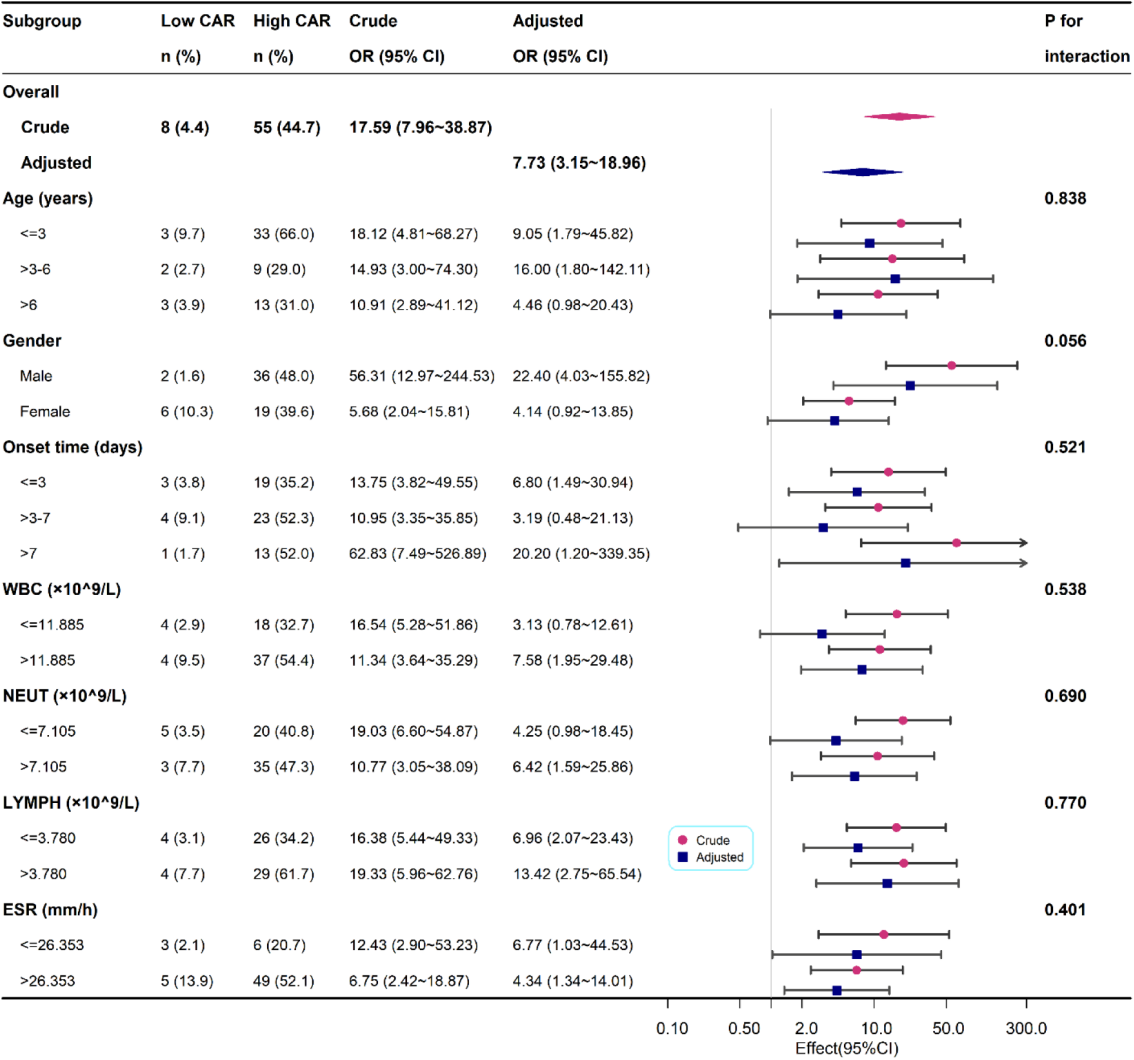
Association between high CAR and PSA according to baseline characteristics. Each stratification adjusted for all the factors except the stratification factor itself.

We also performed a subgroup analysis based on age, gender, onset time, WBC, NEUT, LYMPH and ESR. In the subgroup analysis, we did not observe any significant interaction in the subgroups (all *p* values for interaction were >0.05). Results of the subgroup analysis are shown in the odds-ratio forest plots in Figure 3.

### 3.3 Sensitive analysis

After excluding 29 patients with missing data, a total of 276 patients were analyzed. Univariate analysis using logistic regression model revealed that high CAR (OR = 17.84, 95% CI: 7.67–41.51; *p* < 0.001) was shown to be an independent predictive factor of PSA (Supplementary Table S1). Multivariate logistic regression analysis also indicated that high CAR (OR = 6.62, 95% CI: 1.96–22.28; *p* = 0.002) was a predictor of PSA.

After propensity-score matching (PSM), 39 pairs of each group were finally well-matched (Supplementary Figure S2). There were no significant differences between the two matched groups (Supplementary Tables S2). Among the 39 propensity-matched pairs, the incidence of PSA was significantly higher in the high CAR group [14 (35.9%) vs. 4 (10.3%)]. The results of univariate analysis showed that high CAR (OR = 4.90, 95% CI: 1.44–16.66; *p* = 0.011) was associated with PSA (Supplementary Table S3). In multivariable logistic regression, high CAR (OR = 13.21, 95% CI: 1.12–155.34; *p* = 0.040) was also associated with PSA.

## 4 Discussion

PSA is a very serious condition that can lead to physeal injury and growth arrest and has a devastating consequence if not diagnosed early (6, 20–23). Unfortunately, a single gold standard test for diagnosing PSA does not exist (24). Our purpose was to identify simple and low-cost biomarkers for the early diagnosis of PSA (7). Thus, we assessed retrospectively the diagnostic performance of CAR, NLR, PLR, PVR, and AGR (blood-based biomarkers that are easily obtained from routine laboratory tests in current clinical practice), and then compared their diagnostic values with those of the traditional biomarkers. Besides the convenience and minimal expense necessary, Zareifar et al. found that these biomarkers are generally useful for the diagnosis of infection (25). To compare the diagnostic performance of novel biomarkers, the ROC curves, which are usually used as a measure of the performance of diagnostic test, and the AUC values of these laboratory indicators were calculated. A higher AUC value of a biomarker indicates a higher diagnostic value for PSA.

To our best knowledge, this study is the first cohort on the association between CAR and PSA. In this retrospective study, we found that CAR was independently associated with PSA. By excluding 29 patients with missing data, this result remained robust in the comparisons after PSM. Our findings demonstrated that CAR may be a more sensitive predictive factor in patients with PSA when it is defined by a cut-off level of 0.447 (Table 1). In ROC analysis, the AUC value of CAR was 0.828, corresponding to a sensitivity of 0.873 and a specificity of 0.719. Notably, CAR had the highest AUC (0.828) among all inflammation-based scores, and its AUC was even greater than those of CRP (0.824) which was the best haematological indicator reported by previous studies (26). CRP remained the most significant and independent predictor of septic arthritis (27). The benefit of CRP in identifying septic arthritis was previously proposed by Levine et al. who measured CRP in 133 patients with joint effusion, of which 39 were classified as septic arthritis (28). CRP proved a better independent predictor of PSA than ESR and showed a significant positive correlation with the severity of PSA. Similarly, our study also showed that CRP was found to have better discriminative capability than ESR and WBC in the diagnosis of PSA. Cordemans et al. found that the release of inflammatory mediators lead to an increase in vascular permeability, which promote the leakage of ALB in the inflammatory response (29). Therefore, we hypothesized that CAR might be a better prognostic factor than CRP or albumin alone in PSA. CAR has been used in several published studies as a surrogate for CRP in severity assessments of the inflammatory response (15, 30). In our current finding, CRP increases and ALB decreases in children with septic arthritis. As an alternative novel indicator of inflammation, CAR showed a good correlation with CRP and could have better diagnostic and predictive value for PSA than CRP. Of course, the clinical features should also be considered seriously by pediatric surgeons, personal experience combined with clinical exam may be more effective in determining the condition.

Furthermore, early diagnosis of PSA is important, as it may guide appropriate antibiotic administration and intravenous fluid resuscitation prior to surgical intervention (31). CAR upon admission could guide preoperative antibiotic selection, with the best cut-off point of CAR being 0.447. Although PSA protocols vary widely among countries and regions, 75% of cases can be cured with conservative treatment of antibiotics if symptoms are present for less than 4 days (7). There was a favorable prognosis according to some studies when drainage and antibiotic therapy was initiated within 5–7 days of the onset of septic arthritis (32–35). Conversely, children with PSA recognized on admission typically receive a combined antibiotic therapy, undergo operative treatment, and continue antibiotic therapy postoperatively.

There are several noteworthy limitations of our study. First, potential and unknown confounders may exist, as with any retrospective study. We adjusted for a large number of possible confounders and minimized the influence of these factors that may lead to outcome bias through PSM analysis. Second, there was an inevitable limitation in this study that the assessment of haematological parameters may be influenced by the significant difference of symptom duration. Third, this analysis was a retrospective study and conducted in one hospital with a small sample, further prospective research in a larger cohort is necessary to validate the usefulness of CAR.

## 5 Conclusions

The CAR (>0.447) at admission was an independent risk factor for PSA. It is worthy to further investigate this association.

## Data Availability

The raw data supporting the conclusions of this article will be made available by the authors, without undue reservation.
