# Supplementary material for: The use of the ratio of C-reactive protein to albumin for the diagnosis of pediatric septic arthritis
Source: Front Pediatr. 2024 Jan 16;11:1308513. doi: 10.3389/fped.2023.1308513 (PMC10824903; doi:10.3389/fped.2023.1308513)
Supplement: Supplementary file 1 [file Table1.docx]

Supplementary Material

# Supplementary Tables

## Supplementary Table S1

| [Supplementary Table S1](https://www.ncbi.nlm.nih.gov/pmc/articles/PMC8039324/#SM1) Univariate and multivariable logistic regression analysis of risk factors for PSA before matching. | | | | | |
| --- | --- | --- | --- | --- | --- |
| Variables | Univariate analysis | |  | Multivariate analysis | |
|  | OR (95% CI) | P value |  | OR (95% CI) | P value |
| Age (years) |  |  |  |  |  |
| ≤3 | 1.000 | – |  | 1.000 | – |
| >3-6 | 0.14 (0.06–0.32) | <0.001 |  | 0.26 (0.07–1.02) | 0.053 |
| >6 | 0.19 (0.09–0.39) | <0.001 |  | 0.34 (0.07–1.70) | 0.19 |
| Gender |  |  |  |  |  |
| Male | 1.000 | – |  | 1.000 | – |
| Female | 1.54 (0.85–2.81) | 0.157 |  | 1.03 (0.42–2.51) | 0.957 |
| Onset time (days) |  |  |  |  |  |
| ≤3 | 1.000 | – |  | 1.000 | – |
| >3-7 | 2.30 (1.17–4.50) | 0.015 |  | 1.80 (0.68–4.77) | 0.238 |
| >7 | 0.93 (0.42–2.07) | 0.856 |  | 0.56 (0.16–2.03) | 0.379 |
| Onset site |  |  |  |  |  |
| Hip | 1.000 | – |  | 1.000 | – |
| Knee | 1.15 (0.55–2.42) | 0.705 |  | 0.48 (0.16–1.39) | 0.174 |
| Other | 2.98 (1.35–6.57) | 0.007 |  | 1.24 (0.36–4.29) | 0.736 |
| WBC | 1.11 (1.06–1.17) | <0.001 |  | 0.09 (0.01–1.85) | 0.119 |
| CRP | 1.02 (1.01–1.02) | <0.001 |  | 0.99 (0.98–1.00) | 0.191 |
| ESR | 1.04 (1.03–1.05) | <0.001 |  | 1.01 (0.99–1.03) | 0.377 |
| Albumin | 0.79 (0.72–0.86) | <0.001 |  | 1.14 (0.85–1.51) | 0.385 |
| Globulin | 1.07 (1.00–1.14) | 0.06 |  | 0.78 (0.52–1.17) | 0.233 |
| NEUT | 1.11 (1.04–1.16) | 0.001 |  | 9.41 (0.49–182.33) | 0.138 |
| LYMPH | 1.21 (1.05–1.40) | 0.007 |  | 9.99 (0.44–229.05) | 0.150 |
| MONO | 8.50 (4.04–17.89) | <0.001 |  | 43.57 (1.73–1094.69) | 0.022 |
| Hemoglobin | 0.91 (0.89–0.94) | <0.001 |  | 0.96 (0.92–1.01) | 0.154 |
| PLT | 1.00 (1.00–1.01) | <0.001 |  | 1.01 (0.98–1.04) | 0.439 |
| MPV | 0.88 (0.65–1.19) | 0.404 |  | 0.90 (0.35–2.28) | 0.818 |
| AGR | 0.11 (0.04–0.30) | <0.001 |  | 0.01 (0.01–2.55) | 0.09 |
| NLR | 1.03 (0.97–1.11) | 0.338 |  | 1.29 (1.02–1.63) | 0.036 |
| PLR | 1.00 (1.00–1.00) | 0.644 |  | 0.99 (0.98–1.00) | 0.119 |
| PVR | 1.04 (1.02–1.06) | <0.001 |  | 0.94 (0.74–1.18) | 0.569 |
| CAR |  |  |  |  |  |
| Low CAR | 1.000 | – |  | 1.000 | – |
| High CAR | 17.84 (7.67–41.51) | <0.001 |  | 6.62 (1.96–22.28) * | 0.002 |
| PSA, pediatric septic arthritis; WBC, white blood cell count; CRP, c-reactive protein; ESR, erythrocyte sedimentation rate; NEUT, neutrophil count; LYMPH, lymphocyte count; MONO, monocytes count; PLT, platelet count; MPV, mean platelet volume; AGR, albumin to globulin ratio; NLR, neutrophil to lymphocyte ratio; PLR, platelet to lymphocyte ratio; PVR, platelet to MPV ratio; CAR, c-reactive protein to albumin ratio.  ^*^Adjusted for age, gender, onset time, onset site, WBC, CRP, ESR, Albumin, Globulin, NEUT, LYMPH, MONO, Hemoglobin, PLT, MPV, AGR, NLR, PLR, PVR. | | | | | |

## Supplementary Table S2

| [Supplementary Table S2](https://www.ncbi.nlm.nih.gov/pmc/articles/PMC8039324/#SM1) Baseline covariates before and after matching | | | | | | | | |
| --- | --- | --- | --- | --- | --- | --- | --- | --- |
| **Variables** | **Level** | **Before Matching** | | |  | **After Matching** | | |
|  |  | **Low CAR** | **High CAR** | **SMD△** |  | **Low CAR** | **High CAR** | **SMD△** |
| n |  | 165 | 111 |  |  | 39 | 39 |  |
| Age (%) | <=3 years | 26 (15.8) | 46 (41.4) | 0.521 |  | 10 (25.6) | 11 (28.2) | 0.052 |
|  | >3-6 years | 72 (43.6) | 26 (23.4) | -0.477 |  | 10 (25.6) | 10 (25.6) | 0.000 |
|  | >6 years | 67 (40.6) | 39 (35.1) | -0.115 |  | 19 (48.7) | 18 (46.2) | -0.054 |
| Gender (%) | female | 52 (31.5) | 44 (39.6) | 0.166 |  | 18 (46.2) | 17 (43.6) | -0.052 |
|  | male | 113 (68.5) | 67 (60.4) | -0.166 |  | 21 (53.8) | 22 (56.4) | 0.052 |
| Onset time (%) | <=3 days | 76 (46.1) | 47 (42.3) | -0.075 |  | 20 (51.3) | 20 (51.3) | 0.000 |
|  | >3-7 days | 40 (24.2) | 41 (36.9) | 0.263 |  | 13 (33.3) | 11 (28.2) | -0.106 |
|  | >7 days | 49 (29.7) | 23 (20.7) | -0.221 |  | 6 (15.4) | 8 (20.5) | 0.127 |
| WBC (mean ± SD) |  | 10.35 ± 3.90 | 13.82 ± 6.67 | 0.520 |  | 12.14 ± 5.83 | 11.93 ± 4.14 | -0.032 |
| ESR (mean ± SD) |  | 12.44 ± 18.23 | 47.92 ± 29.37 | 1.208 |  | 24.90 ± 31.37 | 26.59 ± 21.56 | 0.058 |
| Globulin (mean ± SD) |  | 25.04 ± 3.98 | 26.69 ± 4.50 | 0.365 |  | 26.46 ± 5.14 | 26.45 ± 4.41 | -0.003 |
| NEUT (mean ± SD) |  | 5.96 ± 3.51 | 9.06 ± 5.62 | 0.550 |  | 7.70 ± 5.27 | 7.71 ± 4.33 | 0.001 |
| LYMPH (mean ± SD) |  | 3.51 ± 1.61 | 3.53 ± 2.31 | 0.008 |  | 3.24 ± 1.84 | 3.14 ± 1.88 | -0.043 |
| MONO (mean ± SD) |  | 0.63 ± 0.29 | 1.05 ± 0.62 | 0.687 |  | 0.82 ± 0.42 | 0.84 ± 0.36 | 0.025 |
| Hemoglobin (mean ± SD) |  | 126.26 ± 9.81 | 114.39 ± 12.42 | -0.956 |  | 120.72 ± 10.32 | 122.08 ± 9.18 | 0.109 |
| PLT (mean ± SD) |  | 333.15 ± 98.38 | 388.14 ± 151.09 | 0.364 |  | 341.46 ± 103.30 | 343.95 ± 123.54 | 0.016 |
| MPV (mean ± SD) |  | 9.82 ± 1.03 | 9.70 ± 0.93 | -0.121 |  | 9.85 ± 1.03 | 9.73 ± 0.98 | -0.133 |
| AGR (mean ± SD) |  | 1.82 ± 0.27 | 1.60 (0.32 | -0.685 |  | 1.70 ± 0.30 | 1.69 ± 0.30 | -0.032 |
| NLR (mean ± SD) |  | 2.30 ± 3.32 | 3.91 ± 4.14 | 0.389 |  | 3.68 ± 5.94 | 3.57 ± 2.64 | -0.026 |
| PLR (mean ± SD) |  | 109.63 ± 56.23 | 144.31 ± 92.90 | 0.373 |  | 132.58 ± 87.84 | 129.72 ± 46.93 | -0.031 |
| PVR (mean ± SD) |  | 34.78 ± 12.59 | 40.86 ± 17.38 | 0.350 |  | 35.34 ± 12.18 | 36.18 ± 14.61 | 0.048 |
| PSA (%) |  | 7 (4.2) | 49 (44.1) | – |  | 4 (10.3) | 14 (35.9) | – |
| △SMD: standardized mean difference. | | | | | | | | |

## Supplementary Table S3

| [Supplementary Table S3](https://www.ncbi.nlm.nih.gov/pmc/articles/PMC8039324/#SM1) Univariate and multivariable logistic regression analysis of risk factors for PSA after matching. | | | | | |
| --- | --- | --- | --- | --- | --- |
| Variables | Univariate analysis | |  | Multivariate analysis | |
|  | OR (95% CI) | P value |  | OR (95% CI) | P value |
| Age (years) |  |  |  |  |  |
| ≤3 | 1.00 | – |  | 1.00 | – |
| >3–6 | 0.29 (0.06–1.30) | 0.105 |  | 0.06 (0.00–2.22) | 0.127 |
| >6 | 0.38 (0.11–1.27) | 0.115 |  | 1.48 (0.03–77.66) | 0.848 |
| Gender, Female | 1.31 (0.46–3.76) | 0.618 |  | 0.39 (0.03–4.84) | 0.465 |
| Onset time (days) |  |  |  |  |  |
| ≤3 | 1.00 | – |  | 1.00 | – |
| >3–7 | 1.33 (0.40–4.45) | 0.640 |  | 0.77 (0.05–11.06) | 0.849 |
| >7 | 1.60 (0.40–6.45) | 0.509 |  | 0.05 (0.00–1.79) | 0.099 |
| Onset site |  |  |  |  |  |
| Hip | 1.00 | – |  | 1.00 | – |
| Knee | 1.23 (0.38–3.96) | 0.725 |  | 1.24 (0.13–12.07) | 0.854 |
| Other | 4.11 (0.71–23.86) | 0.115 |  | 1197.85 (3.03–473700.73) | 0.020 |
| WBC | 1.03 (0.93–1.14) | 0.565 |  | 0.00 (0.00–0.81) | 0.047 |
| ESR | 1.02 (1.00–1.04) | 0.106 |  | 1.01 (0.97–1.05) | 0.647 |
| Globulin | 1.03 (0.92–1.14) | 0.629 |  | 0.52 (0.21–1.28) | 0.155 |
| NEUT | 1.01 (0.91–1.13) | 0.834 |  | 1082218.13 (0.93–1262202032518.01) | 0.051 |
| LYMPH | 1.15 (0.88–1.50) | 0.321 |  | 2950491.22 (0.99–8774375472716.10) | 0.050 |
| MONO | 4.86 (1.26–18.74) | 0.022 |  | 58309611.99 (15.00–226594193632603.00) | 0.021 |
| Hemoglobin | 0.94 (0.89–1.00) | 0.040 |  | 0.85 (0.72–1.00) | 0.050 |
| PLT | 1.00 (1.00–1.01) | 0.123 |  | 1.00 (0.91–1.11) | 0.941 |
| MPV | 0.70 (0.40–1.24) | 0.222 |  | 0.77 (0.06–9.24) | 0.839 |
| AGR | 0.33 (0.05–2.09) | 0.240 |  | 0.00 (0.00–8.05) | 0.101 |
| NLR | 1.01 (0.91–1.13) | 0.819 |  | 1.09 (0.42–2.81) | 0.865 |
| PLR | 1.00 (0.99–1.01) | 0.841 |  | 1.00 (0.94–1.07) | 0.889 |
| PVR | 1.04 (1.00–1.08) | 0.079 |  | 0.96 (0.41–2.26) | 0.924 |
| CAR |  |  |  |  |  |
| Low CAR | 1.00 | – |  | 1.00 | – |
| High CAR | 4.90 (1.44–16.66) | 0.011 |  | 13.21 (1.12–155.34) ^*^ | 0.040 |
| ^*^ Adjusted for age, gender, onset time, onset site, WBC, ESR, Globulin, NEUT, LYMPH, MONO, Hemoglobin, PLT, MPV, AGR, NLR, PLR, PVR. | | | | | |

# Supplementary Figures

## Supplementary Figure S1


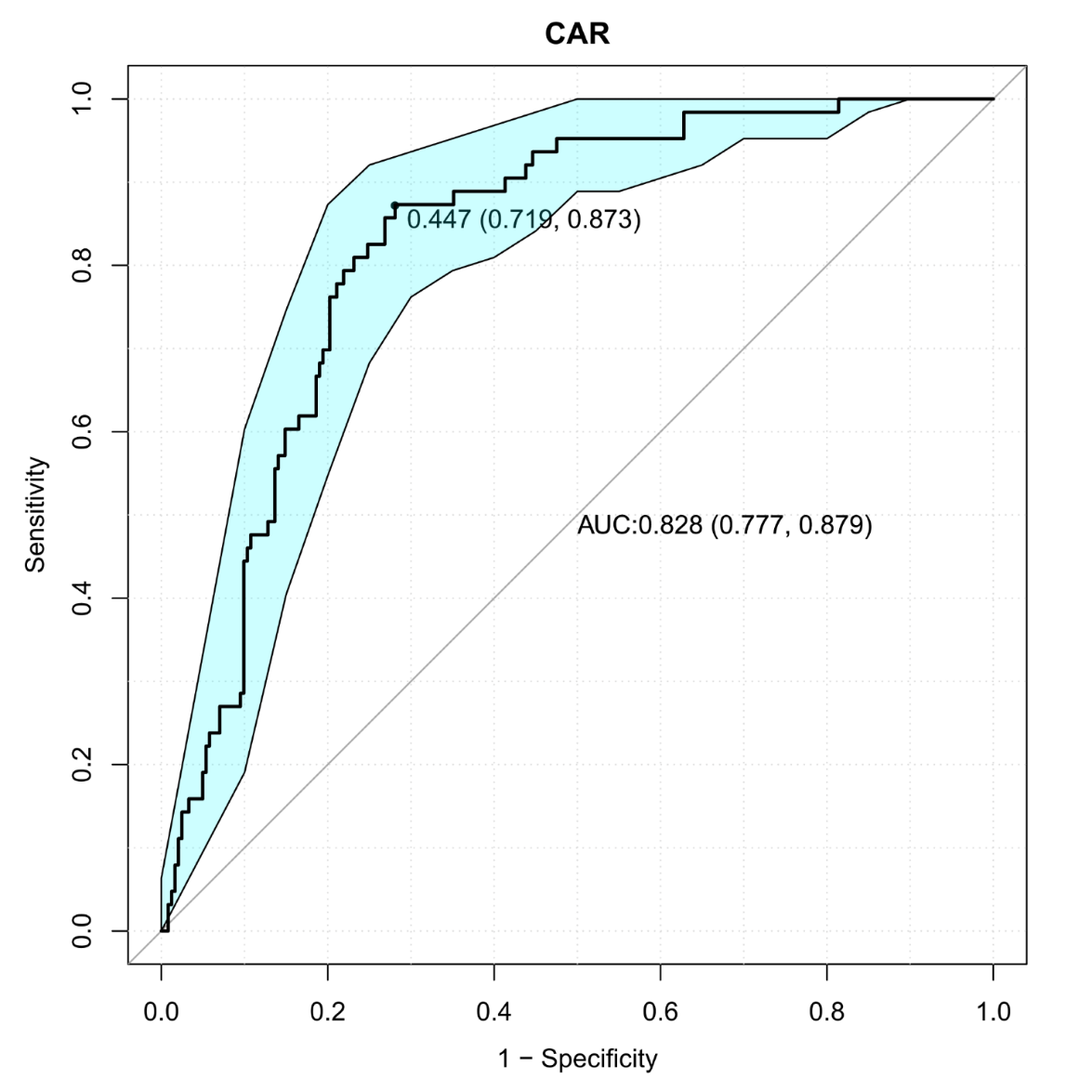


Supplementary Figure S1 Receiver operating characteristic (ROC) curve for the diagnosis of PSA based on CAR.

## Supplementary Figure S2


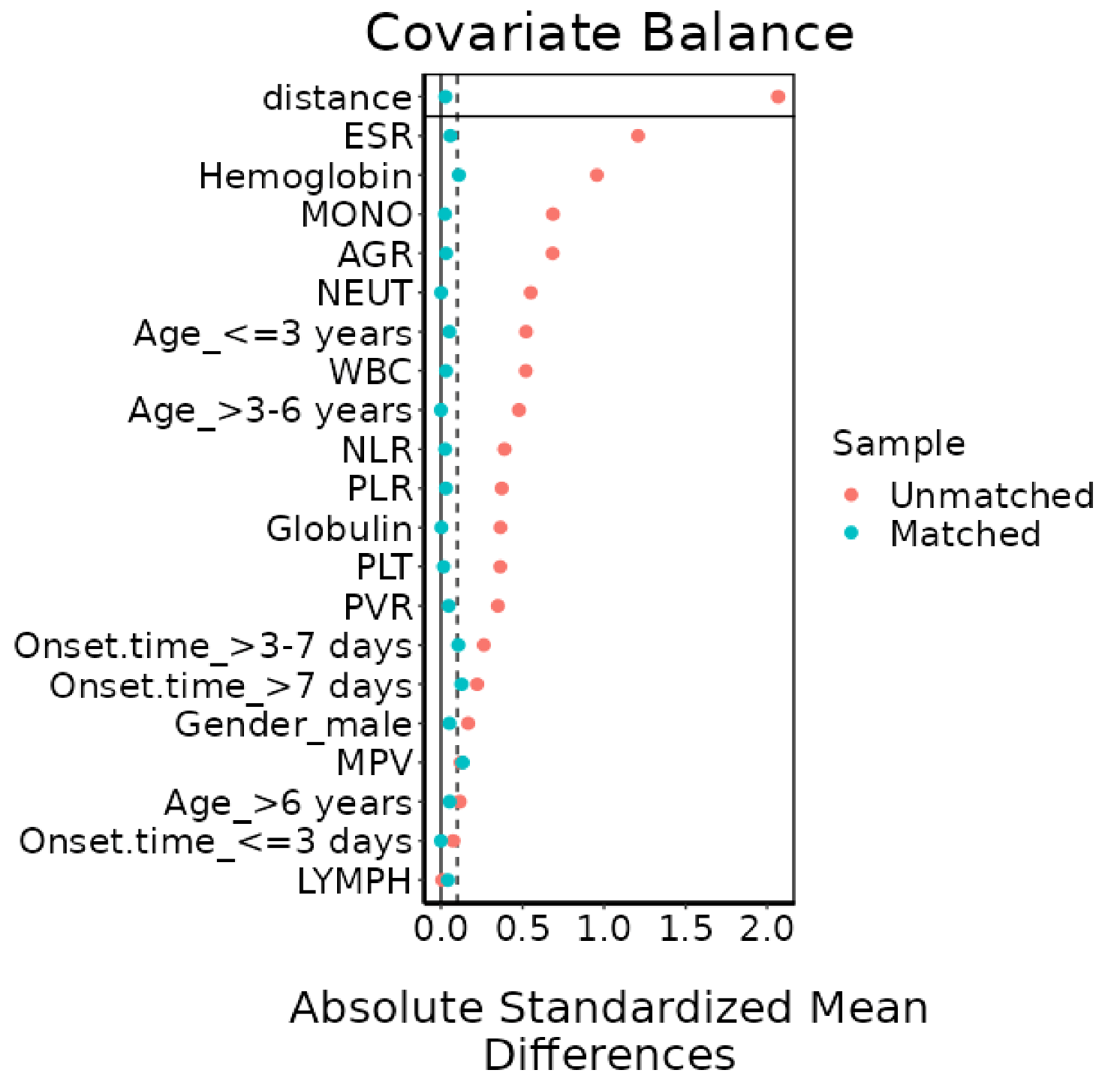


Supplementary Figure S2 Baseline covariates before and after matching.
